# Sensitive Detection of *Escherichia coli* O157:H7 in Food Products by Impedimetric Immunosensors

**DOI:** 10.3390/s18072168

**Published:** 2018-07-05

**Authors:** Francesca Malvano, Roberto Pilloton, Donatella Albanese

**Affiliations:** 1 Department of Industrial Engineering, University of Salerno, Via Giovanni Paolo II, 132, 84084 Fisciano (SA), Italy; fmalvano@unisa.it; 2 Institute of Crystallography of the National Council of Research (CNR), 00015 Monterotondo Scalo, Italy; roberto.pilloton@cnr.it

**Keywords:** label-free immunosensor, *Escherichia coli* 0157:H7, electrochemical impedance spectroscopy

## Abstract

In this work, the development of an impedimetric label-free immunosensor for the detection of *Escherichia coli* O157:H7 is reported. Different immobilization techniques of monoclonal anti-*E. coli* were tested, in order to reach the very low limit of detections. The comparison between the immobilization procedures underlined the advantages of the oriented procedure and the use of a dendrimer, which allowed for immobilizing a higher number of antibody units, reaching a very high sensitivity. However, the use of activated ferrocene as electron-transferring mediator, which improved the electrical properties of the system, resulted in a very low limit of detection equal to 3 cfu/mL. This immunosensor was used to analyze milk and meat samples obtaining a good agreement with the results of the ELISA methods.

## 1. Introduction

Foodborne bacterial pathogens are believed to be the most frequently occurring hazard in the nation’s food and water supply. *Escherichia coli* O157:H7 has emerged as one of the deadliest foodborne pathogens because of its combination of harmfulness and pathogenicity [1].

Illness caused by this microorganism range from bloody diarrhea to life-threatening conditions, such as hemorrhagic colitis, hemolytic uremic syndrome and thrombotic thrombocytopenic purpura. The Centre for Disease Control and Prevention estimates that there are 20,000 illnesses per year due to *E. coli* O157:H7 infection [2]. 

Conventional methods for *E. coli* O157:H7 detection include multiple-tube fermentation (MTF), membrane filter (MF), plate count; although these methods are very sensitive and selective, they are laborious and time-consuming, requiring hands-on preparation and 24 to 48 h of incubation time, which is not practical considering the short shelf-life and cost of storage of the food products [3].

Consequently, detection of pathogens such as bacteria by a rapid, sensitive and cost-effective method is, therefore, a subject of great interest in the field of food industry, environmental monitoring, clinical and diagnostic analysis. 

Nowadays, biosensors, in particular immunosensors, play a significant role in the determination of pathogens, most of them use labeled antibodies to monitor the formation of the antigen-antibody complex [4,5,6].

However, label-free immunosensors, in which the immune interaction between antibody and antigen is directly monitored, exhibit some important advantages in terms of speed and simplicity of operation. Several approaches concerning the developed of label-free immunosensors for the detection of the pathogenic strain *E. coli* O157:H7 have been reported in literature: these include different transduction techniques, such as Surface Plasmon Resonance (SPR) [7], Quartz Crystal Microbalance (QCM) [8], and Surface Acoustic Wave [9]. 

Among all the possible immunosensors (optical, microgravimetric, electrochemical), the electrochemical ones rank highly owing to their sensitivity, low cost and simplicity. Thanks to Electrochemical Impedance Spectroscopy (EIS), food biosensor analysis is performed in real time by studying the change in electrical properties of the electrode surface, which depends only on the binding interaction between the analyte and its receptor [10]. 

Different impedimetric label-free immunosensors for the detection of *E. coli* bacteria have already been developed [11,12,13], while few studies have been conducted on the specific detection of the pathogenic strain *E. coli* O157:H7. Chowdhury [14] immobilized polyclonal anti-*E. coli* on a conductive polyaniline film, reaching detection limits of 10^2^ cfu/mL, but interfering signals were reached with *Salmonella typhi* and *E. coli* BL21 cells. The lowest limit of detection (2 cfu/mL) was reported by Barreiros dos Santos [15] where anti*-E. coli* antibodies have been immobilized onto gold electrodes via self-assembled monolayer of mercaptohexadecanoic acid: a larger linear range (10^1.47^–10^4.47^ cfu/mL) was achieved, but no tests on real food matrices were performed.

In order to detect *E. coli* O157:H7 pathogen in food products, a highly sensitive and selective label-free impedimetric immunosensors have been developed in this study, immobilizing monoclonal anti-*E. coli* O157:H7 (Ab) on gold electrodes functionalized through different immobilization schemes. The performance of all design as impedimetric immunosensor upon binding of *E. coli* cells to the electrode was evaluated by measuring the electron-transfer resistance by EIS in presence of K_3_[Fe(CN)_6_]/K_4_[Fe(CN)_6_] as redox probes. Finally, the immunosensor developed with the immobilization scheme that showed the lowest limit of detection was used to analyze milk and meat samples inoculated with *E. coli* O157:H7 cells.

## 2. Materials and Methods

### 2.1. Chemicals

Ferrocene carboxylic acid (>97%), Cysteamine (95%), Glutaraldehyde solution (50% in H_2_O), Potassium hexacyanoferrate (III) (K_3_[Fe(CN)_6_], >99%), Polyamidoamine (PAMAM) dendrimer generation 4 (ethylenediamine core), were purchased from Sigma-Aldrich (Milano, Italy). Potassium ferrocyanide (K_4_[Fe(CN)_6_]), was obtained from Carlo Erba reagent (Milano, Italy). 4-mercaptobenzoic acid (MBA, 99%), 2-(*N*-morpholino)ethanesulfonic acid (MES > 99.5% purity), *N*-Hydroxysucciminide (NHS, 99%), *N*-(3-Dimethylaminopropyl)-*N*′-ethylcarbodiimide hydrochloride (EDC, >99%), Sulfuric acid (H_2_SO_4_, 99.9%), Ethanolamine (EtNH_2,_ NH_2_CH_2_CH_2_OH, >99.5%), Ethanol (>99.8%) were purchased from Sigma-Aldrich (Milano, Italy), while Protein A/G (5 mg/mL, 59.7 kDa, >98%) was obtained from BioVision Inc., (San Francisco, CA, USA). The *E. coli* O157:H7 antibody (1.4 mg/mL) was purchased from Fitzgerald, while the *E. coli* O157:H7 (heat-killed) was received from SeraCare (Gaithersburg, MD, USA). Sodium phosphate monobasic (NaH_2_PO4), Sodium phosphate dibasic anhydrous (Na_2_HPO_4_), and Potassium Chloride (KCl) used in the preparation of phosphate buffered saline (PBS: 0.1 M KCl, pH 7.4) were obtained from Sigma Aldrich (Milano, Italy).

### 2.2. Apparatus

The electrochemical measurements were carried out with a computer-controlled Autolab PGSTAT 204 Potentiostat (Metrohm, Herisau, Switzerland) equipped with an Impedance module (FRA32M); the experimental data were analyzed with Nova software (Metrohm, The Netherlands). The Au thin-film three-electrode layout (working/auxiliary/reference electrodes) and the “all-in-one” cell were purchased from Micrux Technologies (Oviedo, Spain). The diameter of Au working electrode was 1 mm.

### 2.3. Immunosensors Manufacturing

Before modification, gold electrodes were cleaned by applying different cycles between −1.0 and +1.3 V with 100 mV/s scan rate in 0.05 M sulfuric acid.

Different immobilization schemes were adopted for the immunosensors development:

(a) Not oriented anti-*E. coli* O157:H7 on MBA self-assembled monolayer (Au-MBA-Ab);
(b) Oriented anti-*E. coli* O157:H7 on MBA self-assembled monolayer (Au-MBA-ProteinA/G-Ab);
(c) Anti-*E. coli* O157:H7 on electrochemically deposited cysteamine layers (Au-Cys-Ab);
(d) Anti-*E. coli* O157:H7 on cysteamine and ferrocene layers (Au-Cys-Ferrocene-Ab);
(e) Anti-*E. coli* O157:H7 on PAMAM and ferrocene layers (Au-Cys-PAMAM-Ferrocene-Ab).

The immobilization of Ab in a non-oriented and oriented way (a and b schemes) was carried out according to the procedure described by Malvano et al. [10]. Briefly, a constant potential of 1.2 V for 20 min was applied to the gold electrode dropped with 30 mM MBA ethanol solution; then, the electrode was dipped in a solution of 75 mM EDC and 15 mM NHS in 100 mM MES buffer (pH 7.4) for 2 h.

In the oriented immobilization method (b), Protein A/G 5 mg/mL was dropped on the modified electrode and left to react for 1 h. After incubation, ethanolamine solution was used to block unreacted active sites. In the not oriented immobilization, the antibody was added after the activation of carboxylic groups with EDC/NHS. Then, the electrode was rinsed in PBS to remove unbound antibodies and finally the unreacted active sites were blocked with ethanolamine solution. 

For the immobilization Schemes (c), (d), (e), the surface of the gold electrode was modified with cysteamine layers by electrodeposition [16,17].

For Scheme (c), glutaraldehyde solution 5% (*v*/*v*) was dropped onto the cysteamine modified working electrode for 1 h. After that, the modified electrode was covered with 10 μL of Ab solution (1.4 ng/mL) for 30 min at room temperature. Finally, the unreacted active sites were blocked with 1 M ethanolamine. At the end of each step, the electrode was rinsed in PBS.

For Scheme (d), ferrocene carboxylic acid solution was at first activated with a solution of 75 mM EDC and 15 mM NHS in 100 mM MES buffer (pH 7.4) for 2 h; then, it was dropped on cysteamine modified electrode for 1 h. After that, cysteamine 20 mM was dropped overnight on the surface of the electrode. The link between cysteamine and Ab (1.4 ng/mL) was carried out using glutaraldehyde solution 5% (*v*/*v*). Finally, ethanolamine (1 M) was used to block the unreacted active sites. At the end of each step, the electrode was rinsed in PBS.

For Scheme (e), PAMAM dendrimers (2 mg/mL) were immobilized on cysteamine modified electrode using glutaraldehyde. After that, activated ferrocene (as previously described) was incubated on modified PAMAM electrode surface for 1 h. Finally, the immobilization of Ab in presence of PAMAM dendrimer was carried out by glutaraldehyde 5% (*v*/*v*) and finally dropping on the surface of the modified electrode, Ab solution (1.4 ng/mL) for 30 min at room temperature. The blocking of the unreacted active sites was carried out with 1 M ethanolamine. At the end of each step, the electrode was rinsed in PBS.

**Figure 1:**
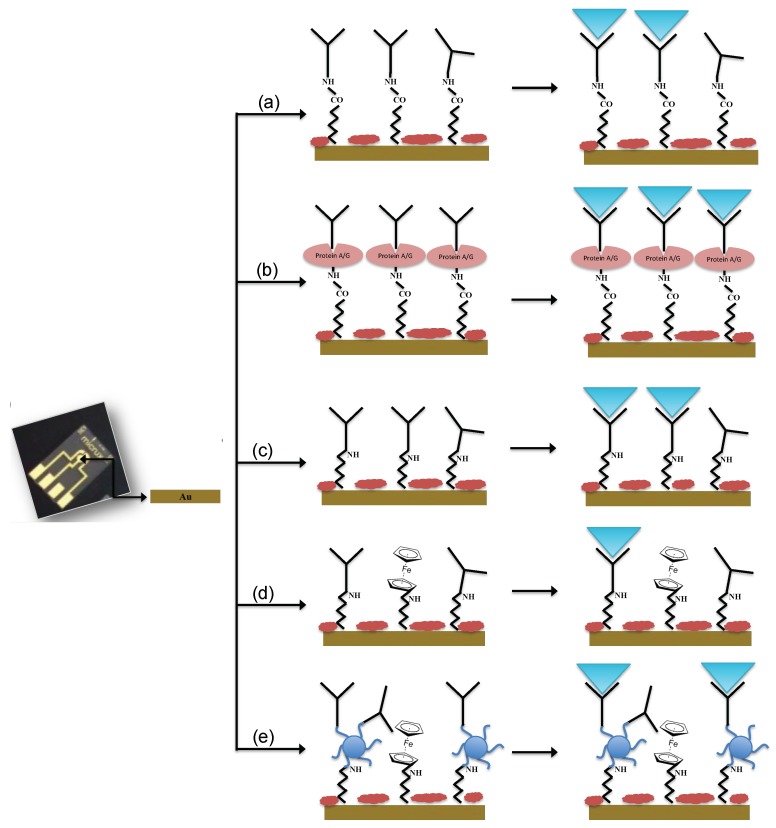
Schematic diagrams of immunosensors fabrication: (**a**) Not oriented anti-*E. coli* O157:H7 on MBA self-assembled monolayer (Au-MBA-Ab); (**b**) Oriented anti-*E. coli* O157:H7 on MBA self-assembled monolayer (Au-MBA-ProteinA/G-Ab); (**c**) Anti-*E. coli* O157:H7 on electrochemically deposited cysteamine layers (Au-Cys-Ab); (**d**) Anti-*E. coli* O157:H7 on cysteamine and ferrocene layers (Au-Cys-Ferrocene-Ab); (**e**) Anti-*E. coli* O157:H7 on PAMAM and ferrocene layers (Au-Cys-PAMAM-Ferrocene-Ab).

The schematic diagrams of the immunosensors fabrication are presented in Figure 1.

### 2.4. Experimental Measurement

EIS was used to characterize each step of the electrode modification and the interactions antibody-*E. coli* O157:H7 heat-killed cells.

For electrochemical impedance studies, a sinusoidal AC (Alternating Current) potential (10 mV) in the frequency range from 0.1 to 10^5^ Hz was superimposed to 0.00 mV DC (Direct Current) potential (on working electrode vs. reference electrode); the measurements were performed in a solution of 1 mM ferri/ferrocyanide redox couple (K_3_[Fe(CN)_6_]/K_4_[Fe(CN)_6_], 1:1) in PB, 0.1 M pH 7, as background electrolyte, at room temperature.

The impedance data were plotted in the form of Nyquist plots, where the complex impedance is displayed as the sum of the real and imaginary components (Z^I^ and Z^II^, respectively) and in the form of Bode diagram, where the total impedance of the system (Z) is plotted versus frequency. Experimental spectra were fitted with a proper equivalent circuit using the facilities of FRA32M (Nova Software).

Cyclic Voltammetry measurements (CV) were also used to monitor the layer by layer construction of the immunosensors design: the measurements were performed from −0.6 to 0.6 V vs. reference electrode with a scan rate of 0.05 V/s; the redox couple used for the CV was the same as that used for impedance measurements.

For *E. coli* measurements, 1 mL of *E. coli* cells at different concentrations were dropped onto the electrode working area and incubated for 90 min; before the impedance measurement, the immunosensors were rinsed with a copious amount of PB.

### 2.5. Preparation of Food Samples for E. coli Detection

Twenty-five grams of meat samples were digested for 2 min at 120 rpm in 250 mL of PBS. The sample was filtered. This procedure was applied to three different *E. coli* concentrations spiked to ground samples in order to obtain 10^3^ cfu/mL, 10^3.70^ cfu/mL and 10^4^ cfu/mL.

As regards the milk sample, 25 mL of milk properly spiked, was mixed in 250 mL of PBS and then it was spiked with the same three different *E. coli* concentrations used for meat samples. 

The results obtained with the immunosensor were compared with those measured with *E. coli* O157:H7 Test Kit (Bioo Scientific, Austin, TX, USA) for *E. coli* detection.

## 3. Results

### 3.1. Design and Comparison of Different Immobilization Layers: CV and EIS Characterizations

In the fabrication of a biosensor, the method of antibodies immobilization on a conductive surface plays a crucial role in the analytical performance of the immunosensor.

The surface modifications of the Au electrodes for the preparation of an *E. coli* immunosensor was monitored using Cyclic Voltammetry (CV) and Electrochemical Impedance Spectroscopy (EIS), which provide useful information on the electrical changes of the electrode surface behaviour after each assembly step.

**Figure 2:**
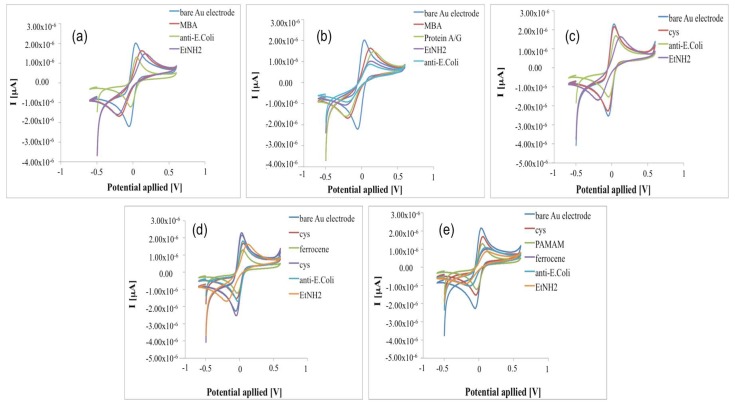
Cyclic Voltammograms in 1 mM K_3_[Fe(CN)_6_]/K_4_[Fe(CN)_6_] after each step of immunosensors construction for all immobilization schemes tested in this work: (**a**) Au-MBA-Ab; (**b**) Au-MBA-ProteinA/G-Ab; (**c**) Au-Cys-Ab; (**d**) Au-Cys-Ferrocene-Ab; (**e**) Au-Cys-PAMAM- Ferrocene-Ab.

The voltammograms of Au electrodes display well defined anodic and cathodic peaks due to the reversible interconversion of the redox probe K_3_[Fe(CN)_6_]/K_4_[Fe(CN)_6_]. After the Au electrodes were covered with short-chain thiols of MBA (immobilization Schemes a and b—Figure 2a,b) and cysteamine (immobilization Schemes c, d and e—Figure 2c–e), the response current decreases due to the MBA and cysteamine layers, which hinder the redox probe on the electrode surface [10,18].

**Figure 3:**
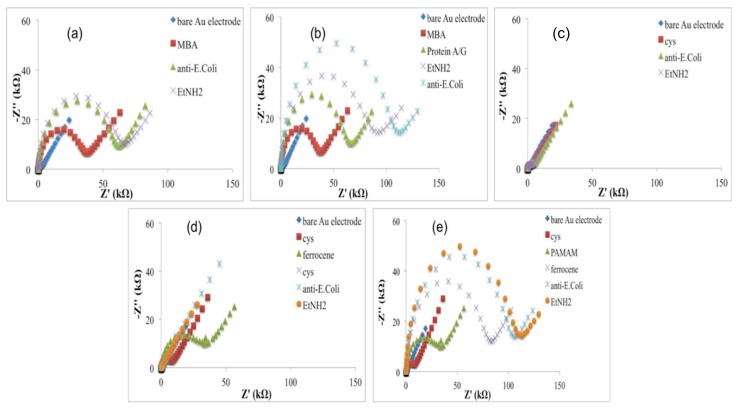
EIS responses to all immunosensor fabrication steps for all immobilization procedures tested in this work: (**a**) Au-MBA-Ab; (**b**) Au-MBA-ProteinA/G-Ab; (**c**) Au-Cys-Ab; (**d**) Au-Cys-Ferrocene-Ab; (**e**) Cys-PAMAM-Ferrocene-Ab.

According to these results, Nyquist plots showed an increase of the impedance, higher for the electrode modified with MBA (Figure 3a,b) than cysteamine (Figure 3c–e), probably due to higher hindering effects of the thiols layer in the electron transfer rate.

In the immobilization Schemes (d) and (e), ferrocene was used to act as an electron-transferring mediator to shuttle electrons between the electroactive probe and electrode surface; thanks to its conductivity, it should improve the electric properties of the system, with the aim to reach very low detection limits [19]. When higher conductivity due to ferrocene did not occur, we thought that the access of the redox probe to the electrode was restricted by the high surface density of ferrocene itself. This hypothesis was confirmed by treating the electrode surface (Au-Cys-Ferrocene) once again with Cys. Because cysteamine directly bonded on the gold surface (Au-Cys) is in equilibrium with cysteamine in solution, we obtain the following substitutions on the Au surface:

*Au-Cys* + *Cys(sol)* ⇆ *Au-Cys* + *Cys (i)*

*Au-Cys-Ferrocene* + *Cys(sol)* ⇆ *Au-Cys* + *Cys-Ferrocene(sol) (ii)*

with the effect to decrease the surface density of ferrocene on Au electrode surface obtaining that ferrocene works as an electron transferring mediator with higher yields than before.

In order to verify this hypothesis, the efficiency of immobilised ferrocene was calculated the Faradaic charge (Q) required for the full oxidation of the immobilized layer; this value is found from the integration of the area under either the anodic or the cathodic peak, corrected from the background current, measured at a slow potential scan rate [20]. The value of Q increased from 3.98 × 10^−5^ C to 3.19 × 10^−4^ C corresponding to *Au-Cys-Ferrocene* before and after the second treatment with cysteamine solution (reaction 2). The increase of the charge can be explained by the highest density in ferrocene before its partial substitution with Cysteamine molecules and thus by the high amount that is probably enough to restrict the access of the redox probe to the electrode, confirmed by a slight increase of the electron transfer resistance (Figure 3d). In this condition, not all the ferrocene molecules, immobilised on the electrode surface, can be reached by the red-ox couple, and, for this reason, ferrocene acted as a transferring electron mediator with a lower yield. According to reactions (i) and (ii), the ferrocene density immobilised on the electrode surface decreased with the effect of an increase of the ferrocene really involved in the electrode reactions. In this case, ferrocene shows its electron-conductive properties, acting as an electron-transferring mediator and causing an impedance decrease (Figure 3d).

Exploiting the electrocatalytic properties of ferrocene, in the configuration (e), a fourth generation PAMAM dendrimer (having 64 surface amines) was used as a linker for the antibody: when PAMAM was loaded on cysteamine/ferrocene-modified electrode, the current response decreased (Figure 2e) with an increase of the impedance (Figure 3e), suggesting that dendrimer molecules, correctly immobilized on modified electrode surface, act as a diffusion barrier for the redox couple and finally to the electron transfer rate.

### 3.2. Immunosensors’ Analytical Performances

**Figure 4:**
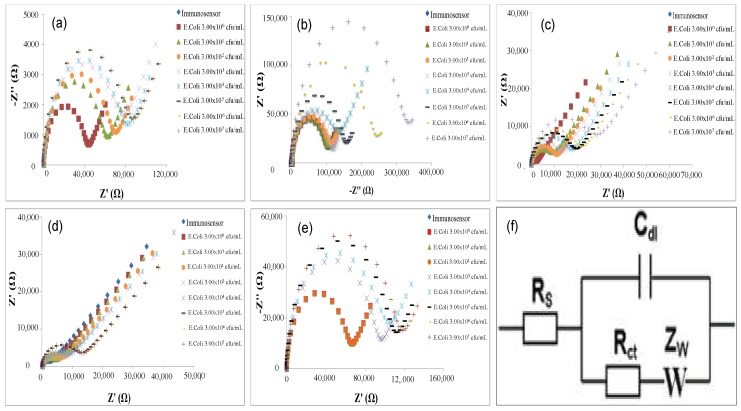
Nyquist plots in impedance measurements before and after the interaction of the immunosensors with different *E. coli* concentrations, for all immobilization procedures tested in this work: (**a**) Au-MBA-Ab; (**b**) Au-MBA-Protein A/G-Ab; (**c**) Au-Cys-Ab; (**d**) Au-Cys-Ferrocene-Ab; (**e**) Cys-PAMAM-Ferrocene-Ab; (**f**) Randle’s Circuit.

When the developed immunosensors react with increasing concentration of *E. coli* cells, in all cases, an increase of semicircle diameters of Nyquist plots was observed (Figure 4), which correspond to the charge transfer resistance R_ct_ of the common Randle’s Circuit used for data fitting (Figure 4f).

This parameter was used to characterize the analytical performance of the fabricated immunosensors; in particular, ∆***R**_ct_* was plotted to calculate the calibration curves, versus the logarithmic value of the *E. coli* cells concentration. ∆***R**_ct_* values were calculated by the following equation:

$$\Delta R_{ct}=R_{ct\left(E.coli\right)}-R_{ct\left(AntiE.coli\right)},$$

where $R_{ct\left(AntiE.coli\right)}$ is the value of the electron transfer resistance when anti-*E. coli* is immobilized on the electrode surface and $R_{ct\left(E.coli\right)}$ is the value of the electron transfer resistance after the bind between antibody-*E. coli* cells.

**Figure 5:**
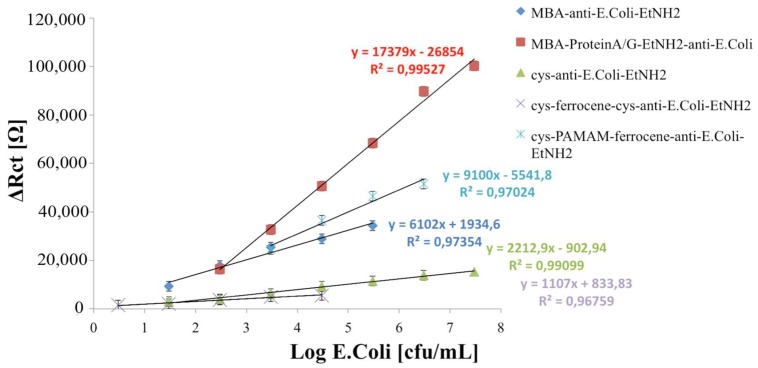
Calibration curves of immunosensors developed; data represent the average values of five immunosensors.

The calibration curves of all developed immunosensors are shown in Figure 5.

The analytical characterization of the immunosensors developed on MBA-modified electrodes versus a different amount of *E. coli* cells (Figure 5) showed a closer linear range but a lower detection limit (LOD), calculated using the sum of average blank solution and three times the standard deviation: 10^1.47^ cfu/mL for the non-oriented immunosensor versus 10^2.47^ cfu/mL for oriented one. This means that, during the random immobilization, a lower number of antibodies molecules is effectively exposed to *E. coli* cells interaction and that, as usual, the receptor-based biosensors decrease their LOD when the effectively immobilized receptor molecules are optimally minimized in order to still obtain a signal from the transducer [10]. In addition, from the analysis of the acquired data, we can hypothesize that, when ferrocene is immobilized, the increased conductivity of the modified electrode surface allows for appreciating smaller variations of impedance, thus improving the resulting LOD for the biosensor.

The comparison between the immunosensors developed on cysteamine layer (Scheme c) and cysteamine/ferrocene layer (Scheme d) highlights the capability of ferrocene molecules to improve the electrical response of the system, reaching a lower limit of detection (10^0.47^ cfu/mL for Scheme d against 10^1.47^ cfu/mL for Scheme c).

Moreover, the immobilization network containing a PAMAM dendrimer, with its numerous advantages related to structural homogeneity and high density of identical functional chain end groups [21], allowed for immobilizing a higher number of Ab, resulting in a significantly higher sensitivity, but in a very close linear range (10^3.47^–10^6.47^ cfu/mL). 

On the basis of the above, all of the developed immunosensors show a good analytical performance: the choice of the optimal immobilization scheme depends on the use of the biosensor (high sensitivity or low limit of detection).

Finally, the reproducibility, calculated on five different immunosensors at 10^3.47^ cfu/mL *E. coli*, showed a good relative standard deviation for all immunosensors developed (Table 1). 

The comparison of the analytical performances of label-free impedimetric immunosensors developed in this study with other recent impedimetric label-free immunosensor for *E. coli* O157:H7 detection is reported in Table 1.

The immunosensor developed with antibodies immobilization on cysteamine/ferrocene (Scheme d) showed a wide linear range and the lowest LOD when compared with the previous impedimetric label-free immunosensors for *E. coli* O157:H7 reported in the literature.

### 3.3. Analysis of E. coli in Food Samples

The immunosensors developed with Schemes a, c and d were used to analyse meat and milk samples spiked with four different *E. coli* concentrations; the results were compared with those obtained from a commercial ELISA kit for *E. coli* detection. The sample preparation used was the same for both analytical methods. The results are shown in Table 2.

The immunosensors’ responses exhibited a good recovery percentage, highlighting the potential of proposed immunosensors as highly capable analytical devices for fast *E. coli* measurement in food products. 

## 4. Conclusions

Different anti-*E. coli* immobilization procedures were tested in this work, in order to develop a new label-free impedimetric immunosensor for the *Escherichia coli* O157:H7 detection in food products.

The comparison between the immobilization procedures analyzed underlines the advantage of the oriented procedure and the use of a PAMAM dendrimer, which allows for immobilizing a higher number of antibody units, reaching a very high sensitivity, but also the use of activated ferrocene as electron-transferring mediator, which improve the electrical properties of the system, resulting in a better impedimetric response.

However, the lowest limit of detection of 3 cfu/mL was obtained in the *E. coli* immunosensors developed on cysteamine/ferrocene-modified electrode: a good agreement between ELISA official methods and this immunosensor was obtained in *E. coli* cells analyzed in food samples.

## Figures and Tables

**Table 1:** Comparing study between the present work with some recent impedimetric label—free immonosensors for *E. coli* O157:H7 detection.

Schematic Immunosensor Assembly	Sensitivity [Ω mL/cfu*cm^2^]	Linear Range [cfu/mL]	LOD [cfu/mL]	RSD [%]	References
Au + PANI + Glu + anti-*E.coli*		1 × 10^2^–1 × 10^7^	100		[14]
Au + MHDA + anti-*E.coli*	772,050.00	3 × 10^1^–3 × 10^4^	20	2.0	[15]
Au-MBA-Ab	784,615.38	3 × 10^1^–3 × 10^5^	30	4.3	This work
Au-MBA-ProteinA/G-Ab	2,228,076.92	3 × 10^2^–3 × 10^7^	300	5.1	This work
Au-Cys-Ab	283,589.74	3 × 10^1^–3 × 10^7^	30	2.9	This work
Au-Cys-Ferrocene-Ab	141,923.07	3 × 10^0^–3 × 10^5^	3	3.8	This work
Au-Cys-PAMAM-Ferrocene-Ab	1,166,666.67	3 × 10^3^–3 × 10^6^	3000	4.7	This work

PANI: Polyaniline; Glu: Glutaraldheyde]; MHDA: 16-mercaptohexadecanoic acid; Cys: Cysteamine; PAMAM: Polyamidoamine dendrimer; Ab: antibody.

**Table 2:** *E. coli* results in spiked milk and meat samples obtained by impedimetric immunosensors and ELISA kit.

Sample	Spiked Concentration [cfu/mL]	Immunosensors						ELISA Kit	
		Scheme a [cfu/mL]	Recovery [%]	Scheme c [cfu/mL]	Recovery [%]	Scheme d [cfu/mL]	Recovery [%]	Found Concentration [cfu/mL]	Recovery [%]
Milk	1.00 × 10^2^	0.97 × 10^2^	97.00	0.92 × 10^2^	92.00	0.97 × 10^2^	97.00	1.05 × 10^2^	105.00
	1.00 × 10^3^	0.91 × 10^3^	91.00	0.95 × 10^3^	95.00	0.98 × 10^3^	98.00	1.07 × 10^3^	107.00
	5.00 × 10^3^	4.90 × 10^3^	98.00	4.93 × 10^3^	98.60	4.95 × 10^3^	99.00	4.79 × 10^3^	95.80
	1.00 × 10^4^	0.91 × 10^4^	91.00	0.94 × 10^4^	94.00	0.96 × 10^4^	96.00	1.13 × 10^4^	113.00
Meat	1.00 × 10^2^	0.95 × 10^2^	95.00	0.93 × 10^2^	93.00	0.99 × 10^2^	99.00	1.01 × 10^2^	101.00
	1.00 × 10^3^	0.98 × 10^3^	98.00	0.93 × 10^3^	93.00	0.96 × 10^3^	96.00	0.87 × 10^3^	98.00
	5.00 × 10^3^	5.25 × 10^3^	105.00	5.09 × 10^3^	101.80	5.05 × 10^3^	99.00	4.17 × 10^3^	95.00
	1.00 × 10^4^	0.96 × 10^4^	96.00	0.97 × 10^4^	97.00	0.94 × 10^4^	94.00	1.05 × 10^4^	95.00
